# The Relationship Between Burnout, Depression, and Anxiety: A Systematic Review and Meta-Analysis

**DOI:** 10.3389/fpsyg.2019.00284

**Published:** 2019-03-13

**Authors:** Panagiota Koutsimani, Anthony Montgomery, Katerina Georganta

**Affiliations:** Department of Educational & Social Policy, School of Social Sciences, Humanities and Arts, University of Macedonia, Thessaloniki, Greece

**Keywords:** burnout, depression, anxiety, meta-analysis, systematic review

## Abstract

**Background:** Burnout is a psychological syndrome characterized by emotional exhaustion, feelings of cynicism and reduced personal accomplishment. In the past years there has been disagreement on whether burnout and depression are the same or different constructs, as they appear to share some common features (e.g., loss of interest and impaired concentration). However, the results so far are inconclusive and researchers disagree with regard to the degree to which we should expect such overlap. The aim of this systematic review and meta-analysis is to examine the relationship between burnout and depression. Additionally, given that burnout is the result of chronic stress and that working environments can often trigger anxious reactions, we also investigated the relationship between burnout and anxiety.

**Method:** We searched the online databases SCOPUS, Web of Science, MEDLINE (PubMed), and Google Scholar for studies examining the relationship between burnout and depression and burnout and anxiety, which were published between January 2007 and August 2018. Inclusion criteria were used for all studies and included both cross-sectional and longitudinal designs, published and unpublished research articles, full-text articles, articles written in the English language, studies that present the effects sizes of their findings and that used reliable research tools.

**Results:** Our results showed a significant association between burnout and depression (*r* = 0.520, SE = 0.012, 95% CI = 0.492, 0.547) and burnout and anxiety (*r* = 0.460, SE = 0.014, 95% CI = 0.421, 0.497). However, moderation analysis for both burnout–depression and burnout–anxiety relationships revealed that the studies in which either the MBI test was used or were rated as having better quality showed lower effect sizes.

**Conclusions:** Our research aims to clarify the relationship between burnout–depression and burnout–anxiety relationships. Our findings revealed no conclusive overlap between burnout and depression and burnout and anxiety, indicating that they are different and robust constructs. Future studies should focus on utilizing more longitudinal designs in order to assess the causal relationships between these variables.

## Introduction

One of the most common psychological symptoms modern people increasingly experience is burnout, i.e., the outcome of chronic, work-related stress (Melamed et al., 2006). Burnout descriptions can be found in the historical record and they appear to be apparent across different times and cultures (reports of burnout feelings can be found from the Old Testament to Shakespeare's writings) (Kaschka et al., 2011). However, it was not until the mid 1970s that researchers have started investigating burnout feelings. In particular, two independent researchers, Herbert Freudenberger, a psychiatrist, and Christina Maslach, a social psychologist, were the first researchers who began examining burnout. Specifically, Freudenberger (1974) was the first to describe the concept of staff burnout. The basic elements of his definition of burnout described these experiences as to fail, wear out, or become exhausted by making excessive demands on energy, strength or resources, and can still be seen in the modern definitions of job burnout. Maslach et al. (1996) defined burnout as the experience of exhaustion, where the individuals who suffer from it become cynical toward the value of their occupation and doubt their ability to perform. According to Maslach et al. (1996), burnout is composed of three dimensions i.e., exhaustion, cynicism, and lack of professional efficacy. In more particular, exhaustion refers to feelings of stress, specifically chronic fatigue resulting from excessive work demands. The second dimension, depersonalization or cynicism, refers to an apathetic or a detached attitude toward work in general and the people with whom one works; leading to the loss of interest in work, and feeling that work has lost its meaning. Finally, lack of professional efficacy refers to reduced feelings of efficiency, successful attainment, and accomplishment both in one's job and the organization.

As Maslach and Leiter (2016) later highlighted, burnout is the result of prolonged interpersonal stressors at work. Research has shown that burnout is related to reduced performance in the workplace (Ruotsalainen et al., 2015) often leading to several forms of withdrawal, such as absenteeism and intention to leave the job (Alarcon, 2011; Kim and Kao, 2014). To put it in other words, it is the worker's inability or lack of resources to meet with the demands that are associated with the job tasks (Weber and Jaekel-Reinhard, 2000; Maslach et al., 2001; Bianchi et al., 2015a). It has been argued, for instance, that burnout is not only associated with difficulties related to the working environment, but also other factors, such as learned helplessness, learning theory, environmental and/or personality factors (for a review see Kaschka et al., 2011). To quote Bühler's and Land's (2003) question “why under the same working conditions one individual burns out, whereas another shows no symptoms at all?” we need to keep in mind that burnout is in fact a response to stressful events (Cherniss, 1980) and how each individual responds to such events depends on how he/she evaluates them (Sarason, 1972; Lazarus and Folkman, 1984); therefore, a person's reaction to a work stressor might range from minor to significant stimulation. In other words, while there are employees who report that they experience burnout, there are others who do not, although they all work within the same working environment. A possible mechanism that differentiates employees' reaction to a stressful working environment is personality characteristics. Personality can either be a coping mechanism which allows individuals to acquire/conserve resources and protect themselves from deviant behavior (Ghorpade et al., 2007) or it can make someone more susceptible and vulnerable to stressors. Two crucial psychological phenomena which are related with personality, are depression and anxiety. As Middeldorp et al. (2006) mention neuroticism, i.e., emotional instability and proneness to anxiety (Eysenck and Rachman, 2013), and low extraversion are positively correlated with both depression and anxiety. Indeed, emotional stability has been shown to be negatively related to the core component of burnout, i.e., emotional exhaustion, and depersonalization and positively related to personal accomplishment (Ghorpade et al., 2007), whereas extroversion has been found to be negatively related to emotional exhaustion and positively related to personal accomplishment (Ghorpade et al., 2007). That is to say, individuals who are more extroverted and more emotionally stable, are less likely to develop burnout and vice versa. However, the question as to what degree burnout is differentiated from depression and anxiety, or whether they complement each other, remains unanswered; and this question is crucial as burnout might be falsely labeled as depression and/or anxiety disorders, leading to inappropriate treatment techniques.

### Burnout and Depression

There is disagreement among researchers who study burnout as to whether there is an overlap between burnout and depression (Bianchi et al., 2015a). As Freudenberger (1974) mentions, people who suffer from burnout look and act as if they were depressed. Indeed, we cannot overlook the fact that some of the burnout symptoms appear to resemble the ones of depression; as it is characterized by anhedonia, i.e., the loss of interest or pleasure, depressed mood, fatigue or loss of energy, impaired concentration, and feelings of worthlessness, decreased or increased appetite, sleep problems (hypersomnia or insomnia) and suicidal ideation (American Psychiatric Association, 2013). However, despite its severity and resemblance to depression characteristics, burnout is not mentioned in DSM-V and still no diagnostic criteria exist for identifying it (Bakusic et al., 2017). It is worth noting that in clinical practice, exhausted employees are being diagnosed with burnout and frequently, in order for the clinicians to proceed with their treatment, they turn to alternative diagnoses like the ones of depression or exhaustion (Kaschka et al., 2011). Yet, the question is still an open one, to what degree can we differentiate burnout from depression and anxiety?

Bianchi and Brisson (2017), for instance, examined to what extent individuals with burnout and depression attribute their feelings to their job. What the researchers found was that the number of the participants who attributed their burnout feelings to their job was proportional to the ones who attributed their depressive symptoms to their job as well, indicating that there might be an overlap between burnout and depression in relation to their antecedents. Many studies have also shown that there is a positive correlation between burnout and depression (Glass and McKnight, 1996; Schaufeli and Enzmann, 1998; Bianchi et al., 2013, 2014, 2015b; Bianchi and Laurent, 2015). Indeed, as Bianchi et al. (2015a) mention in their systematic review, it has been found that inventories that assess burnout, and more specifically the subscale of emotional exhaustion–the core component of burnout–are positively correlated with depressive symptoms (Takai et al., 2009; Bianchi et al., 2013; Ahola et al., 2014). Several researchers have argued that because studies have found a consistent medium to high correlation between the two concepts, this might suggest an overlap and that burnout might not be a distinct psychological phenomenon but a dimension of depression (Bianchi et al., 2015b). Additionally in terms of consequences, in a recent study by Bianchi et al. (2018a) it was observed that both burnout and depression were associated not only with the increased recall of negative words, but also with the decreased recall of positive words. The authors concluded that burnout and depression overlap with each other and this overlap extends also to emotional memory. It is worth noting, and regarding the diagnostic differentiation between burnout and depression, in their review Kaschka et al. (2011) mention that correlations between burnout and depression appear frequently among relevant studies, showing that either there is an overlap between burnout and depression, or burnout probably might be a risk factor of developing depression. As it regards to the similarity of the two constructs at a biological level, in their systematic review, Bakusic et al. (2017) found that burnout and depression appear to share a common biological basis. In particular, according to the researchers', the epigenetics studies so far appear to advocate toward a probable mediator, i.e., DNA methylation, which might act as a biomarker of stress-related mental disorders, such as depression, burnout and chronic stress. Therefore, we can observe that besides the psychological common characteristics these two constructs appear to share, they also seem to share a common biological basis.

On the other hand, not all researchers seem to agree with the above notion. Although burnout and depression appear to share some common features (e.g., loss of energy), several researchers believe that burnout and depression are two separate constructs (Ahola and Hakanen, 2007) and that emotional exhaustion is not related to depression (Schaufeli and Enzmann, 1998). There are quite a few studies which have shown that burnout and depression do not overlap with each other and that burnout is differentiated from depression (Bakker et al., 2000; Schaufeli et al., 2001; Toker and Biron, 2012). Furthermore, one major factor that appears to distinguish burnout from depression is the fact that burnout is work related and situation specific, whereas depression is context free and pervasive (Freudenberger and Richelson, 1980; Maslach et al., 2001; Iacovides et al., 2003). That is, burnout is specifically related to someone's working environment, while depression can show up regardless of the circumstances of the environment (e.g., social or family environment). Nevertheless, it should be noted that this distinction might not be very accurate as depression at its first stages might be domain specific (Rydmark et al., 2006). Thus, it is plausible that depression might start as work-related stress or it might evolve as burnout, as this work-related stress gets stronger.

The existing literature is still inconclusive as to whether burnout and depression are the same or different constructs and, although most of the research studies are cross-sectional, longitudinal studies also provide mixed results (McKnight and Glass, 1995; Hakanen and Schaufeli, 2012). As Bianchi et al. (2015b) note, the aim of most longitudinal studies is not to examine the casual relationship between the two variables, but they are designed in order to predict whether burnout can predict depression or vice versa. All in all, despite the majority of the research studies that examine the relationship between burnout and depression, we are not still able to answer whether the two phenomena are the same or different constructs. By conducting the present meta-analysis, we aim to provide more clarification concerning this relationship. Additionally, by knowing if burnout in its essence falls under the umbrella of depression diagnosis, it would provide valuable information as to whether it should be included in the diagnostic criteria of depression or it should be integrated as a different diagnostic entity.

### Burnout and Anxiety

One other factor that appears to be related with burnout, but is not as frequently investigated in relation to it as depression, is anxiety (Sun et al., 2012). Anxiety is a common psychological condition which acts as a protective factor against threatening situations (Cole, 2014). However, prolonged anxiety might result in psychological distress affecting an individual's everyday functioning (Cole, 2014). According to Ahmed et al. (2009), anxiety is “a psychological and physiologic state characterized by cognitive, somatic, emotional, and behavioral components.” Nevertheless, although anxiety is considered a general reaction to threatening situations, it is divided into two related constructs; trait and state anxiety (Turnipseed, 1998). In particular, trait anxiety is an individual's stable characteristic and the degree to which he/she perceives stressful situations as threatening, i.e., a person's proneness to anxiety (Spielberger, 1966). On the other hand, state anxiety is the individual's reaction toward a situation after having appraised it as threatening (Spielberger, 1966). That is, an individual's proneness to anxiety reflects trait anxiety, whereas state anxiety is the reaction after a situation has been appraised as threatening. Some researchers suggest that occupational stress might in fact be a risk factor for anxiety symptoms (DiGiacomo and Adamson, 2001; Sun et al., 2012). For example, in the study of Vasilopoulos (2012) the participants who reported high social anxiety levels reported high burnout levels as well. Additionally, Mark and Smith (2012) found that job demands, extrinsic effort, and over-commitment were associated with increased anxiety levels. Similarly, Ding et al. (2014) found that emotional exhaustion and cynicism were positively related to anxiety symptoms, whereas professional efficacy was negatively related to anxiety symptoms. That is, the more emotionally exhausted, cynical, and less efficient toward his/her work an individual feels, the more anxious he/she will be. Turnipseed (1998) also found that burnout and anxiety symptoms are significantly correlated with each other, with the strongest link existing between anxiety and emotional exhaustion. According to Turnipseed (1998), this interaction between work situations and individuals' personalities –as mentioned earlier– creates a state of anxiety and, by extension, contributes to burnout onset. However, to our knowledge it is still unclear the exact relationship between burnout and anxiety. Specifically, are people with higher trait anxiety more prone to developing burnout or do burnout feelings compound anxiety symptoms? Furthermore, is there an overlap between burnout and anxiety?

### Objectives

Overall, the evidence regarding the relation between burnout and depression and burnout and anxiety are still inconclusive. The purpose of this systematic review and meta-analysis is to investigate and clarify the association between the above variables. Our goal is to clarify the existing evidence and have a better understanding of the relationship between burnout and depression and burnout and anxiety.

### Research Questions

Our research questions were the following:

1. Is there an overlap between burnout and depression?
2. Is there a potential moderator underlying the relationship between burnout and depression?
3. Is there an overlap between burnout and anxiety?
4. Is there a potential moderator underlying the relationship between burnout and anxiety?

## Methods

### Systematic Review Protocol

**Figure 1:**
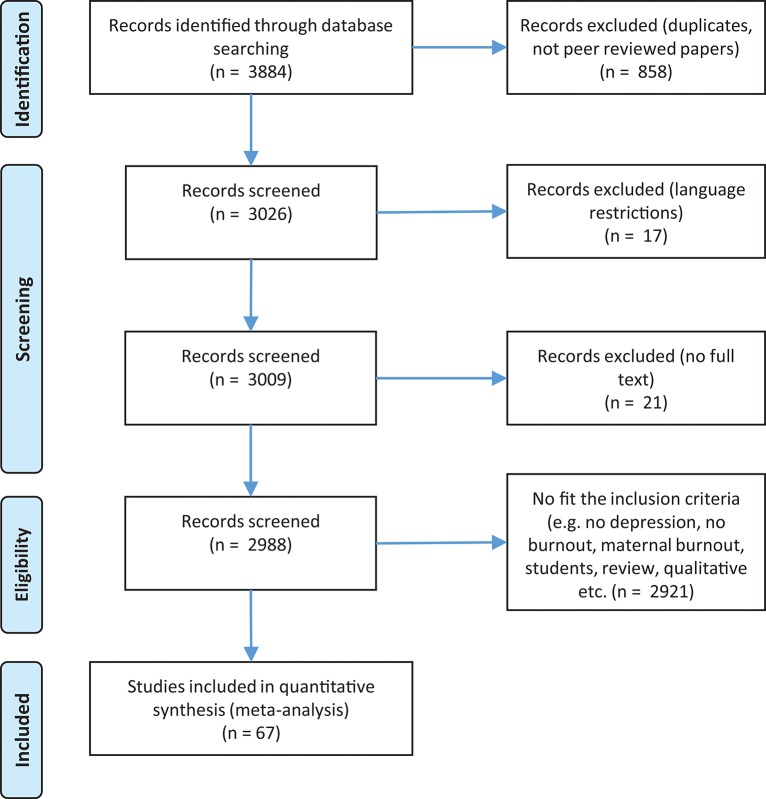
Flow diagram for burnout and depression.

**Figure 2:**
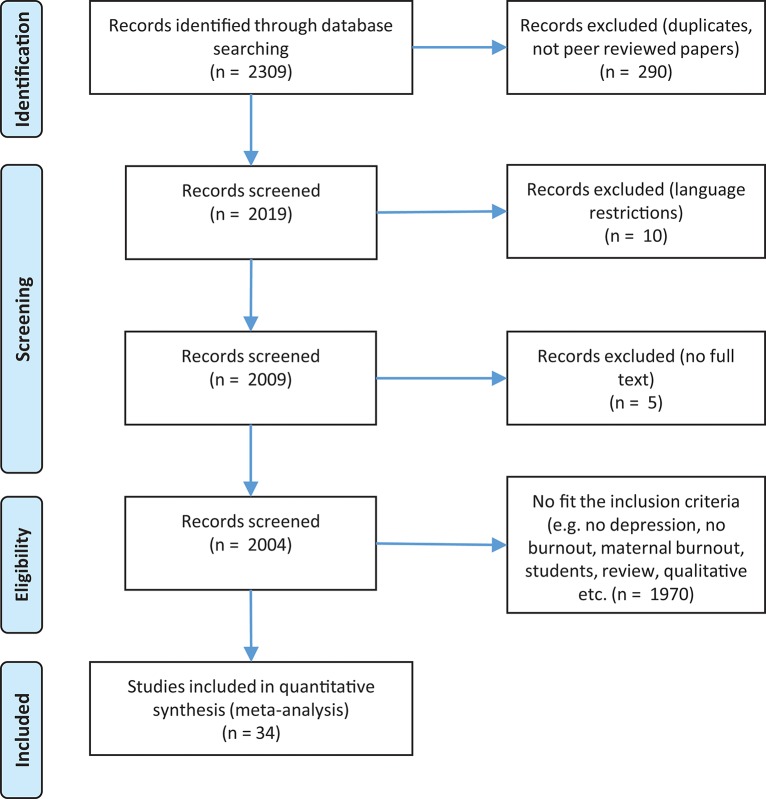
Flow diagram for burnout and anxiety.

Before we began our database search, firstly we searched PROSPERO's database for possible registered protocol reviews that might have been conducting the same meta-analysis. As no such protocol review was found at PROSPERO's database, we wrote and registered a systematic protocol review in which we stated our purpose with the current meta-analysis, our eligibility criteria and our search strategy. After the registration of our systematic protocol review (CRD42018090505), we continued with the database search. Specifically, selection procedure, study identification, and critical appraisal of the research studies was conducted according to the checklist presented in the Preferred Reporting Items for Systematic Reviews and Meta-analyses (PRISMA) statement (Moher et al., 2009; see Figures 1, 2), Supplementary Data Sheet S1. For burnout and depression, 67 papers were identified which resulted in 69 studies for analysis. For burnout and anxiety 34 papers were identified which resulted in 36 studies for analysis.

### Search Strategy

We searched the online databases SCOPUS, Web of Science, MEDLINE (PubMed) and Google Scholar for research published between January 2007 and August 2018. The combinations of the key words we used were the following: *burnout, depression, anxiety*. Additionally, we used MeSH terms with the term “*burnout”* being the major topic of the meta-analysis and our search was formed as follows: *burnout/depression [majr] AND burnout/anxiety [majr]; burnout/depression [majr] OR burnout/anxiety [majr]*. After we completed the electronic database search, a manual scoping of the cited studies by all articles found was also done in case some of them did not show up in our search.

Our eligibility criteria included; (i) all types of studies, both cross-sectional and longitudinal, (ii) published and unpublished research articles, (iii) full-text articles, (iv) research articles written in the English language, (v) studies that present the effects sizes of their results and (vi) studies that used reliable research tools. Additionally, all studies had to describe the types of methods they used in order to assess burnout, depression and anxiety. Regarding the type of the populations used in the studies, we included studies that examined employed individuals and professional athletes as well.

Furthermore, we categorized the research studies into five moderators, depending on the type of the assessment tools that were used and the type of the studies (cross-sectional or longitudinal) in which they were utilized. Specifically, and after we conducted frequencies analyses, it was found that the most widely used tools for assessing our variables of interest were the Maslach Burnout Inventory (MBI) (Maslach et al., 2006) for assessing burnout, the Patient Health Questionnaire (PHQ) for assessing depression (Spitzer et al., 1994; Kroenke et al., 2001) and the Hospital Anxiety Depression Scale (HADS) (Zigmond and Snaith, 1983) for assessing anxiety. Consequently, the three moderator variables that were created were: (i) the MBI vs. Non-MBI studies, (ii) the PHQ vs. Non-PHQ studies, and (iii) the HADS vs. Non-HADS studies. The fourth moderator was the type of the study, i.e., cross-sectional or longitudinal. This way we were able to examine whether the assessment tools and/or the type of the studies had different effect on the results or not. Lastly, the fifth moderator was occupation.

### Quality Assessment

Quality assessment was performed based on the Quality Assessment Tool for Observational Cohort and Cross-Sectional Studies (Feng et al., 2014). The tool contains 14 criteria and the evaluator is asked to answer whether the study in question meets the criterion. The possible answers are Yes, No, Cannot Determine, Not applicable, and Not Reported. A score of >11 corresponds to good quality, 7–10 to fair quality and <7 to poor quality. Of the 67 studies measuring burnout and depression that were included in the meta-analysis 28 were rated by two independent evaluators as fair and 40 as good (one paper contained 2 studies - each one of the two studies was evaluated differently). Of the 34 studies measuring burnout and anxiety that were included in the meta-analysis 15 were rated by two independent evaluators as fair and 19 as good.

### Analysis

All analyses were guided by Lipsey and Wilson (2001) and conducted using Comprehensive Meta-Analysis software (Lipsey and Wilson, 2001; Borenstein et al., 2005). In deriving effect sizes and confidence intervals, random-effects models were used. Random-effects models assume variation in effect sizes between studies, and this is due to both sampling error and true random variance arising from differences between studies in terms of their procedures and settings (as opposed to only sampling error stipulated in a fixed effect model). In comparison to fixed-effects models, then, random-effects models are generally considered to be preferable and allow generalization beyond the set of studies examined to future studies (Schmidt et al., 2009).

The summary statistic reported is the weighted r. Cohen provided rules of thumb for interpreting these effect sizes, suggesting that an r of 0.10, represents a “small” effect size, 0.30 represents a “medium” effect size and 0.50 represents a “large” effect size (Cohen, 1992). However, researchers have suggested that the indiscriminate use of Cohen's generic small, medium, and large effect size values to characterize effect sizes in domains in which normative values do not apply is inappropriate and misleading (Lipsey et al., 2012). Therefore, it is important that effect sizes are grounded in the context by assessing their contribution to knowledge.

### Moderation Analysis

The following moderators were examined as possible reasons for heterogeneity; burnout measure (MBI vs. Non-MBI measurement of burnout), the emotional exhaustion dimension of the MBI vs. the other two dimensions vs. the dimensions of the Non-MBI scales (Emotional exhaustion vs. Non-Emotional exhaustion scales vs. other burnout scales), the depression measure (PHQ vs. Non-PHQ), the anxiety measure (HADS vs. Non-HADS), the type of study (Cross-sectional vs. Longitudinal), the occupational status (Healthcare vs. Educational vs. Other professionals), and their quality as described above (Fair vs. Good quality). The selection of the above measurements as moderators was decided after taking into consideration the frequency in which they were used in the studies.

Moderation was assessed by calculating the degree of inconsistency in the observed relationship across studies (*I*^2^). This index is interpreted as the percentage of total variation across studies due to “true” heterogeneity rather than sampling error (Higgins et al., 2003). As *I*^2^ increases, the level of true heterogeneity increases (0 to 100%). Values of 25, 50, and 75% have been identified as low, medium, and high levels of heterogeneity.

## Results

### Studies Retrieved for the Meta-Analysis

As it regards the number of records that were originally identified, concerning burnout, and depression a total of 3,884 records were found. After refining the search results, 3,026 records were screened, 21 of them were excluded as they were not full-texts, 17 were excluded due to language restrictions (non-English), and 2,921 because they didn't fit the inclusion criteria (e.g., no depression, no burnout, maternal burnout, students, review, qualitative etc.) or were excluded because the appropriate statistics were not provided. In total 67 papers (69 studies) were included in the meta-analysis (see Figure 1).

Concerning burnout and anxiety, 2,309 records were identified. After refining the results, 2,019 available records were screened; 10 of them were excluded due to non-use of the English language, 5 were not full-texts and 1,970 were excluded because they didn't fit the inclusion criteria (e.g., no depression, no burnout, maternal burnout, students, review, qualitative etc.) or because the appropriate statistics were not provided. In total 34 papers (36 studies) were eligible for the meta-analysis (see Figure 2).

### Study Selection and Characteristics

Tables 1, 2 provide a detailed summary of all the studies that were included in the meta-analysis for both depression and anxiety, respectively. In total 101 studies were included in this review; 67 studies for burnout and depression and 34 studies for burnout and anxiety. Table 3 provides a list of all the questionnaires used in the studies included in the meta-analysis (includes the abbreviations).

**Table 1:** Studies measuring burnout and depression included in the meta-analysis (69 studies).

	**Studies (in alphabetical order)**	***n***	**Burnout measure**	**Depression measure**	**Design**
1	Ahola et al., 2014	1,964	MBI-HS	BDI-SF	Longitudinal
2	Bakir et al., 2010	377	MBI	BDI	Cross-sectional
3	Bauernhofer et al., 2018	103	MBI-GS	BDI	Cross-sectional
4	Bianchi and Brisson, 2017	468	SMBM	PHQ9	Cross-sectional
5	Bianchi and Laurent, 2015	54	MBI	BDI-II	Cross-sectional
6	Bianchi and Laurent, 2015	54	BM	BDI-II	Cross-sectional
7	Bianchi and Schonfeld, 2016	323	SMBM	PHQ9	Cross-sectional
8	Bianchi and Schonfeld, 2018	911	MBI-GS	PHQ-8	Cross-sectional
9	Bianchi et al., 2013	1,658	MBI	BDI-II	Cross-sectional
10	Bianchi et al., 2014	5,575	MBI	PHQ-9	Cross-sectional
11	Bianchi et al., 2015b	627	MBI	PHQ-9	Longitudinal
12	Bianchi et al., 2016a	1,046	SMBM	PHQ-9	Cross-sectional
13	Bianchi et al., 2016b	184	SMBM	PHQ-9	Cross-sectional
14	Bianchi et al., 2018a	1,056	SMBM	PHQ-9	Cross-sectional
15	Bianchi et al., 2018b	222	SMBM	PHQ-9	Cross-sectional
16	Bianchi et al., 2018c	1,015	SMBM	PHQ-9	Cross-sectional
17	Capone and Petrillo, 2018	285	MBI-GS	CES-D	Cross-sectional
18	Cardozo et al., 2012	212	MBI-HS	HSCL-25	Longitudinal
19	Choi et al., 2018	386	MBI-GS	PHQ	Cross-sectional
20	Choi et al., 2018		ProQOL	PHQ	Cross-sectional
21	da Silva Valente et al., 2018	1,046	MBI	PHQ-9	Cross-sectional
22	De Stefano et al., 2018	26	MBI	BDI	Cross-sectional
23	Duan-Porter et al., 2018	281	OLBI	PHQ-9	Longitudinal
24	Favrod et al., 2018	208	MBI	HADS	Cross-sectional
25	Fong et al., 2016	312	CBI	HADS	Longitudinal
26	Garrouste-Orgeas et al., 2015	1,534	MBI	CES-D	Cross-sectional
27	Grover et al., 2018	445	MBI	PHQ-9	Cross-sectional
28	Hakanen et al., 2008	2,555	MBI	BDI	Longitudinal
29	Hakanen and Schaufeli, 2012	1,964	MBI	BDI	Longitudinal
30	Hemsworth et al., 2018	273	ProQOL	DASS-21	Cross-sectional
31	Hintsa et al., 2014	3,283	MBI-GS	BDI	Cross-sectional
32	Idris and Dollard, 2014	117	MBI	PHQ-9	Longitudinal
33	Johnson et al., 2017	323	MBI	DASS-21	Cross-sectional
34	Karaoglu et al., 2015	74	MBI	HADS	Cross-sectional
35	Lebensohn et al., 2013	168	MBI	CES-D	Cross-sectional
36	Lee et al., 2018	464	MBI-GS	HADS	Cross-sectional
37	Lobo, 2018	10	MBI	HADS	Cross-sectional
38	Malmberg-Gavelin et al., 2018	119	SMBQ	HADS	Cross-sectional
39	Mather et al., 2016	5,093	PBM	SCID	Cross-sectional
40	Melchers et al., 2015	944	MBI-GS	BDI-II	Cross-sectional
41	Metlaine et al., 2018	140	MBI	HADS	Cross-sectional
42	Moore and Schellinger, 2018	62	PQLS	CES-D	Cross-sectional
43	Mosing et al., 2018	10,120	MBI-GS	SCL-90	Cross-sectional
44	Mutkins et al., 2011	80	MBI	DASS-21	Cross-sectional
45	Oe et al., 2018	158	MBI	HADS	Cross-sectional
46	Penz et al., 2018	412	MBI	PHQ-9	Cross-sectional
47	Pereira-Lima and Loureiro, 2015	400	BSI	PHQ-4	Cross-sectional
48	Peterson et al., 2008	3,719	OLBI	HADS	Cross-sectional
49	Plieger et al., 2015	755	MBI-GS	BDI-II	Cross-sectional
50	Richardson et al., 2018	119	CBI	IDAS-II	Cross-sectional
51	Rogers et al., 2014	349	CBI	PHQ	Cross-sectional
52	Samios, 2017	69	ProQOL	DASS-21	Cross-sectional
53	Santa Maria et al., 2018	811	CBI	PHQ-2	Cross-sectional
54	Schiller et al., 2018	51	SMBM	HADS	Cross-sectional
55	Schonfeld and Bianchi, 2016	1,386	SMBM	PHQ-9	Cross-sectional
56	Silva et al., 2018	100	BSI	PHQ-9	Cross-sectional
57	Steinhardt et al., 2011	267	MBI	CES-D	Cross-sectional
58	Takai et al., 2009	84	PBM	BDI-II	Cross-sectional
59	Talih et al., 2016	118	BM	PHQ-9	Cross-sectional
60	Talih et al., 2018	91	BM	PHQ-9	Cross-sectional
61	Toker and Biron, 2012	1,632	SMBM	PHQ	Longitudinal
62	Tourigny et al., 2010	550	MBI	CES-D	Cross-sectional
63	Trockel et al., 2018	250	PFI	PROMIS	Cross-sectional
64	Tzeletopoulou et al., 2018	72	MBI	CES-D	Cross-sectional
65	van Dam, 2016	113	MBI	SCL-90	Cross-sectional
66	Vasconcelos et al., 2018	91	MBI-HS	BDI	Cross-sectional
67	Weigl et al., 2016	313	MBI	STDS	Cross-sectional
68	Wurm et al., 2016	5,897	HBI	MDI	Cross-sectional
69	Yeh et al., 2018	172	OBI	EPDS	Cross-sectional

**Table 2:** Studies measuring burnout and anxiety included in the meta-analysis (36 studies).

	**Studies (in alphabetical order)**	***n***	**Burnout measure**	**Depression measure**	**Design**
1	Andreassen et al., 2018	988	MBI-GS	GHQ-28	Cross-sectional
2	Bianchi and Laurent, 2015	54	MBI	HADS	Cross-sectional
3	Bianchi and Laurent, 2015	54	BM	HADS	Cross-sectional
4	Bianchi and Schonfeld, 2018	911	MBI-GS	Self-constr.	Cross-sectional
5	Cardozo et al., 2012	212	MBI-HS	HSCL-25	Longitudinal
6	Choi et al., 2018	386	MBI-GS	GAD-7	Cross-sectional
7	Choi et al., 2018	386	ProQOL	GAD-7	Cross-sectional
8	Craiovan, 2015	60	CBI	HAM-A	Cross-sectional
9	De Stefano et al., 2018	26	MBI	STAI	Cross-sectional
10	Demir, 2018	335	BSI-SV	IPIP	Cross-sectional
11	Diestel and Schmidt, 2010	324	MBI	STAI	Cross-sectional
12	Ding et al., 2014	1,243	MBI-GS	SAS	Cross-sectional
13	Favrod et al., 2018	208	MBI	HADS	Cross-sectional
14	Gallego-Alberto et al., 2018	101	MBI	POMS	Cross-sectional
15	Gillet et al., 2018	521	SMBM	JAS	Cross-sectional
16	Hemsworth et al., 2018	273	ProQOL	DASS-21	Cross-sectional
17	Karaoglu et al., 2015	74	MBI	HADS	Cross-sectional
18	Katkat, 2015	336	MBI	SSAI	Cross-sectional
19	Lee et al., 2018	464	MBI-GS	HADS	Cross-sectional
20	Lobo, 2018	10	MBI	HADS	Cross-sectional
21	Malmberg-Gavelin et al., 2018	119	SMBQ	HADS	Cross-sectional
22	Mather et al., 2016	5,093	PBM	SCID	Cross-sectional
23	Metlaine et al., 2018	140	MBI	HADS	Cross-sectional
24	Mutkins et al., 2011	80	MBI	DASS-21	Cross-sectional
25	Oe et al., 2018	158	MBI	HADS	Cross-sectional
26	Pereira-Lima and Loureiro, 2015	400	BSI	PHQ-4	Cross-sectional
27	Peterson et al., 2008	3,719	OLBI	HADS	Cross-sectional
28	Schiller et al., 2018	51	SMBM	HADS	Cross-sectional
29	Shi et al., 2018	696	MBI-GS	Self-constr.	Cross-sectional
30	Talih et al., 2016	118	BM	GAD-7	Cross-sectional
31	Talih et al., 2018	91	BM	GAD-7	Cross-sectional
32	Trockel et al., 2018	250	PFI	PROMIS	Cross-sectional
33	van Dam, 2016	113	MBI	SCL-90	Cross-sectional
34	Yazicioglu and Kizanlikli, 2019	284	MBI	STAI	Cross-sectional
35	Zhou et al., 2016	1,274	MBI	SAS	Cross-sectional
36	Zhou et al., 2018	1,354	MBI	SAS	Cross-sectional

**Table 3:** Questionnaires used for measuring burnout, depression and anxiety in the studies included in the meta-analysis.

**Short name**	**Name**
**BURNOUT**	
BSI	Burnout Syndrome Inventory
CBI	Copenhagen Burnout Inventory
HBI	Hamburg Burnout Inventory
MBI	Maslach Burnout Inventory
OBI	Occupational Burnout Inventory
OLBI	Oldenburg Burnout Inventory
PBM	Pines Burnout Measure
PFI	Professional Fulfillment Index
PQLS	Professional Quality of Life Scale
ProQOL	Professional Quality of Life
SMBM	Shirom–Melamed Burnout Measure
SMBQ	Shirom-Melamed Burnout Questionnaire
**DEPRESSION**	
BDI	Beck Depression Inventory
CES-D	Center for Epidemiologic Studies Depression Scale
DASS-21	Depression Anxiety Stress Scales
EDPS	Edinburgh Postnatal Depression Scale
HADS	Hospital Anxiety and Depression Scale
HSCL-25	Hopkins Symptom Checklist-25
IDAS-II	Inventory of Depression and Anxiety Symptoms-II
MDI	Major Depression Inventory
PHQ	Patient Health Questionnaire
PROMIS	Patient-Reported Outcomes Measurement Information System
SCID	Structured Clinical Interview for DSM-IV Disorders
SCL-90	Symptom Checklist
STDS	State-TraitDepressionScales
**ANXIETY**	
–	1 Item Self-Constructed
DASS-21	Depression Anxiety Stress Scales
GAD-7	Generalized Anxiety Disorder-7
GHQ-28	General Health Questionnaire-28
HADS	Hospital Anxiety and Depression Scale
HAM-A	Hamilton Anxiety Rating Scale
HSCL-25	Hopkins Symptom Checklist-25
IPIP	International Personality Item Pool
JAS	Job-Anxiety-Scale
PHQ-4	Patient Health Questionnaire-4
POMS	Profile of Moods State
PROMIS	Patient-Reported Outcomes Measurement Information System
SAS	Zung Self-Rating Anxiety Scale
SCID	Structured Clinical Interview for DSM-IV Disorders
SCL-90	Symptom Checklist
SSAI	Spielberger State Anxiety Inventory
STAI	State and Trait Anxiety Scales

Concerning the publication year of the studies about burnout and depression, 43.3% of them were published during 2018 (until August), followed by 13.4% of them which were published in 2016 and 11.9% in 2015; 7.5% of the studies were published in 2014, 5.6% in 2017, 4.5% in 2012, 15.6% were published in each of the following years: 2008, 2010, 2011, and 2013 (3.9% each), and 1.5% in 2009. In relation to publication year of the studies about burnout and anxiety, 52.9% were published in 2018 (until August), 11.8% studies were published in 2015, 11.8% in 2016, 5.8% in 2014 and 5.8% 2017; 11.6% were published during the years of 2007, 2010, 2011, and 2012 (2.9% each).

Regarding the studies relating to burnout and depression, the overall sample size for the 67 studies was 84,169 participants, 30,942 (37%) men, and 49,898 (59%) women (three studies did not include gender characteristics). Of the studies that measured burnout, 55% of them used the MBI and variations of it (i.e., MBI-GS, MBI-HS), 14.5% used the SMBM test, 5.8% used the CBI test, 4.3% used the BM, and 4.3% the ProQOL tests, 8.7% of them used the BSI, the OLBI and the PBM tests, and 7% used other measures of burnout (HBI, OBI, PFI, PQLS, and SMBQ); in two studies burnout was measured with two tests, MBI and PBM and MBI and ProQOL test.

Most of the studies (36.1%) used the PHQ to measure depression, 20.2 % of them used the BDI, 14.5% used the HADS, another 10.1% of the studies used the CES-D, 5.8% used the DASS-21, 2.9% used the SCL-90 and, lastly, 1.4% used the EPDS, HSCL-25, IDAS-II, MDI, PROMIS, SCID, and STDS tests.

Respectively, in the studies relating to burnout and anxiety, the overall sample size for the 34 studies were 40,751 participants, 15,561 (38%) were men, and 23,915 (59%) were women (in two studies gender characteristics were not included). Concerning the burnout tool which was used across the studies, 63.9% of them used the MBI test and its variations (i.e., MBI-GS, MBI-HS), 8.3% used the BM test, 5.6% used the BSI, ProQOL and SMBM tests and 2.8% used the BSI, CBI, OLBI, PBM, PFI, and SMBQ tests. In two studies burnout was measured with two tests, MBI and PBM, and MBI and ProQOL test.

In relation to the measurement of anxiety, 30.6% of the studies used the HADS, 11.1% used the GAD-7, 8.3% used the STAI and the SAS, 5.6% of the studies used the DASS-21 and 1-item self-constructed test, and 2.8% of the studies used the GHQ-28, HAM-A, HSCL-25, IPIP, JAS, PHQ-4, POMS, PROMIS, SCID, SCL-90, and SSAI tests.

Concerning the design of the studies, 87% of them examining the burnout and depression relationship utilized a cross-sectional design, and 13% were longitudinal; 97.2% of the studies measuring burnout and anxiety utilized a cross-sectional design and 2.8% were longitudinal.

### Main Meta-Analysis: Association Between Burnout and Depression

**Figure 3:**
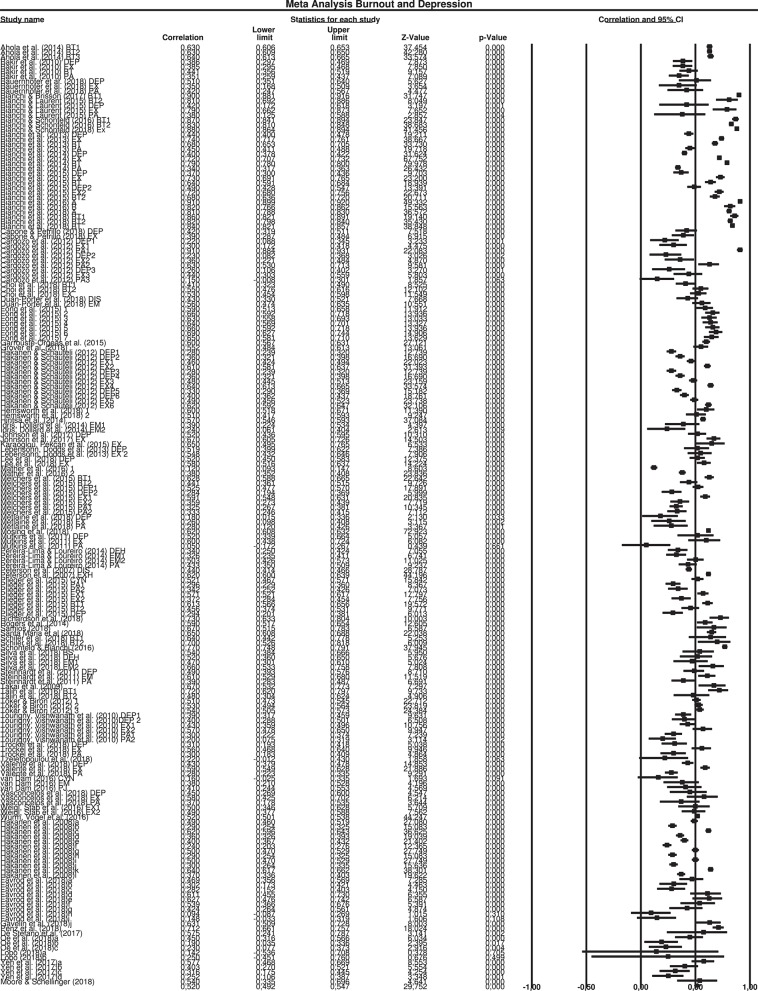
Meta analysis burnout and depression.

Overall results indicated a significant effect (*r* = 0.520, SE = 0.012, 95% CI = 0.492, 0.547). The confidence intervals around the effect sizes for each study are presented in the forest plot (see Figure 3).

### Main Meta-Analysis: Association Between Burnout and Anxiety

**Figure 4:**
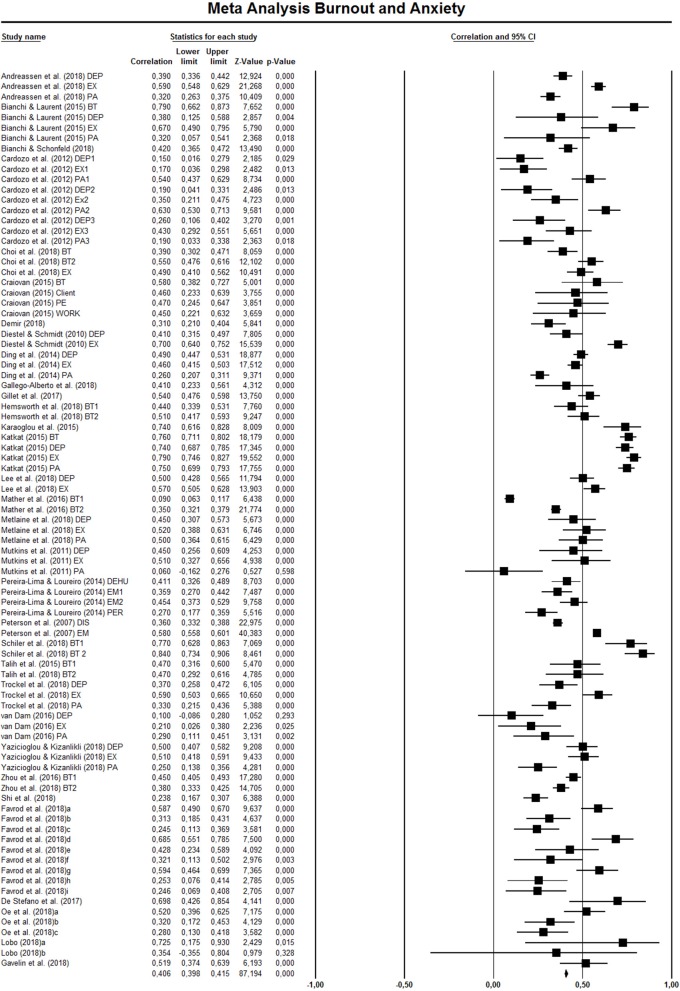
Meta analysis burnout and anxiety.

Overall results indicated a significant effect (*r* = 0.460, SE = 0.014, 95% CI = 0.421, 0.497). The confidence intervals around the effect sizes for each study are presented in the forest plot (see Figure 4).

### Sub-group Analysis: Measure and Context

The meta-analysis indicated significant heterogeneity with the I-squared = 98.432 and I-squared = 95.367 for both depression and anxiety respectively, suggesting that moderation analysis was appropriate.

In terms of context, the depression studies that used the MBI reported lower effect sizes (*r* = 0.472, SE = 0.011, 95% CI = 0.441, 0.503) in comparison with other scales (*r* = 0.622, SE = 0.042, 95% CI = 0.564, 0.675). Likewise, anxiety studies that used the MBI reported slightly lower effect sizes as well (*r* = 0.451, SE = 0.011, 95% CI = 0.406, 0.493) in comparison with other scales (*r* = 0.482, SE = 0.029, 95% CI = 0.408, 0.549).

Concerning the burnout dimension, in the burnout—depression relationship the effect sizes of the emotional exhaustion dimension were higher (*r* = 0.508, SE = 0.012, 95% CI = 0.467, 0.546) comparing to the other dimensions of the MBI test (*r* = 0.409, SE = 0.006, 95% CI = 0.380, 0.437), but lower compared to the other burnout measurements that report total burnout scores (*r* = 0.749, SE = 0.136, 95% CI = 0.643, 0.827) and those that report scores from individual subscales (*r* = 0.608, SE = 0.005, 95% CI = 0.574, 0.639). With respect to the burnout—anxiety relationship, the effect sizes of the emotional exhaustion dimension were slightly higher (*r* = 0.472, SE = 0.012, 95% CI = 0.417, 0.524), compared to the other dimensions of the MBI test (*r* = 0.426, SE = 0.017, 95% CI = 0.369, 0.479) and slightly lower compared to the other burnout measurements that report total scores (*r* = 0.494, SE = 0.060, 95% CI = 0.318, 0.637) and those that report scores from individual subscales (*r* = 0.499, SE = 0.052, 95% CI = 0.379, 0.602).

Additionally, the studies that used the PHQ scale reported higher effect sizes (*r* = 0.628, SE = 0.040, 95% CI = 0.565, 0.684) in comparison with other scales (*r* = 0.481, SE = 0.009, 95% CI = 0.453, 0.507). Likewise, in relation to anxiety, studies that used the HADS scale reported higher effect sizes as well (*r* = 0.507, SE = 0.023, 95% CI = 0.448, 0.562) in comparison with other scales (*r* = 0.437, SE = 0.016, 95% CI = 0.387, 0.484).

With respect to the design of the studies (cross-sectional or longitudinal), concerning the burnout—depression relationship, the cross-sectional studies reported higher effect sizes (*r* = 0.526, SE = 0.022, 95% CI = 0.488, 0.562) comparing to the longitudinal ones (*r* = 0.505, SE = 0.009, 95% CI = 0.466, 0.543). Concerning the burnout—anxiety relationship, sub-group analysis regarding the design of the studies was not conducted as there was only one longitudinal study in the meta-analysis.

As it regards the occupational status, and specifically the burnout—depression relationship, educational staff reported higher effect sizes (*r* = 0.679, SE = 0.049, 95% CI = 0.609, 0.738) comparing to healthcare workers (*r* = 0.495, SE = 0.008, 95% CI = 0.466, 0.524) and the general employed population (*r* = 0.449, SE = 0.020, 95% CI = 0.399, 0.496). Regarding the burnout—anxiety relationship, healthcare professionals reported slightly lower effect sizes (*r* = 0.436, SE = 0.010, 95% CI = 0.396, 0.475) comparing to the general employed population (*r* = 0.492, SE = 0.035, 95% CI = 0.418, 0.559). Sub-group analysis with the educational staff was not conducted as there were only two studies in which the participants fitted in the occupational status.

Lastly, with respect to the quality of the studies (see section Quality Assessment), concerning the burnout—depression relationship, the studies with fair quality reported slightly higher effect sizes (*r* = 0.565, SE = 0.032, 95% CI = 0.515, 0.610) comparing to the good quality studies (*r* = 0.488, SE = 0.009, 95% CI = 0.456, 0.518). Concerning the burnout—anxiety relationship, the studies with fair quality reported slightly higher effect sizes (*r* = 0.466, SE = 0.009, 95% CI = 0.418, 0.511) comparing to the good quality studies (*r* = 0.453, SE = 0.018, 95% CI = 0.402, 0.502).

### Publication Bias

In order to assess publication bias (the “file-drawer” problem) we adopted a number of strategies. We examined the fail-safe number (fail-safe N) for each effect size. We also inspected funnel plots (a scatterplot of effect sizes against the reciprocal of its standard error).

Rosenthal (1979) recommends that the fail-safe number should be >5 k + 10, where k equals the number of observed effect sizes (Rosenthal, 1979). In the present analysis the classic fail-safe *N* is 8,603 and 5,932 for the burnout—depression relationship and the burnout—anxiety relationships, respectively. Rosenthal's method has been critiqued on the grounds that it fails to take into account the bias in the “file drawer” of unpublished studies, and thus can give misleading results (Scargle, 1999). Therefore, we also calculated Orwin's fail-safe N, which was equal to 944 (depression) and 288 (anxiety) (using 0.10 as a criterion for a trivial correlation).

**Figure 5:**
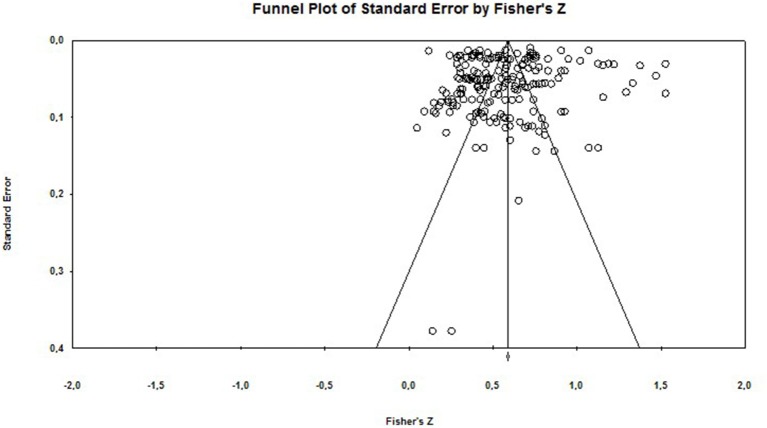
Funnel plot of Standard Error for burnout and depression.

**Figure 6:**
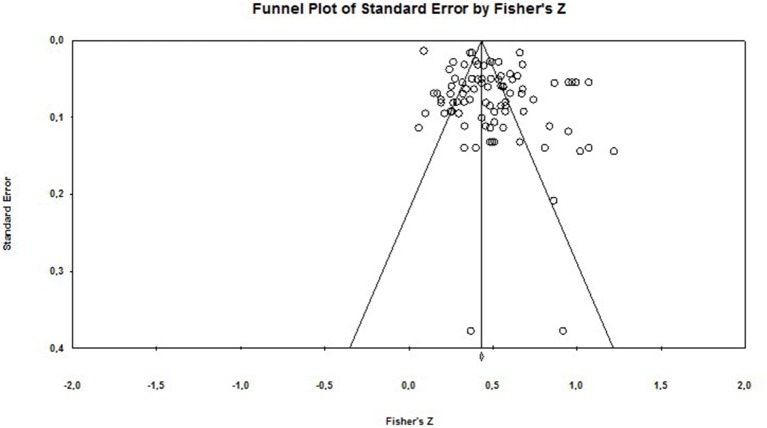
Funnel plot of Standard Error for burnout and anxiety.

In terms of publication bias, the funnel plots (see Figures 5, 6) indicate a degree of asymmetry. However, funnel plots are not a good way to investigate publication bias *per se*, as there can be a number of reasons for asymmetrical funnel plots (also called small study effects), which are due to heterogeneity, reporting bias and poor methodological design (Sterne et al., 2011; Sedgwick, 2013).

## Discussion

### Summary of Main Findings

During the last decade, research regarding the relationship of burnout and depression, and burnout and anxiety, has grown. As we observed from our database search on the studies that measure the aforementioned relationships, the research in this field of area has increased in recent years, with the majority of the studies being conducted during the last year (43.5 and 52.8% for the burnout—depression and burnout—anxiety relationships, respectively). The interest on clarifying these relationships appears to be growing stronger and by conducting the present meta-analysis we wanted to clarify whether there is an overlap between burnout and depression, and an overlap between burnout and anxiety. Overall, burnout research is growing—particularly when it comes to small-scale occupational studies, but the research tends to be varied, and applies a range of different instruments to measure burnout (Eurofound, 2018). It is possible that employees who have been diagnosed with a depressive and/or an anxiety disorder might also suffer from burnout (Eurofound, 2018). Indicatively, Maske et al. (2016) found that 59% of individuals who have been diagnosed with burnout they were also diagnosed with an anxiety disorder, 58% with an affective disorder, i.e., depression or a depressive episode and 27% with a somatoform disorder. In other words, the similarities between burnout and depression and burnout and anxiety might lead to false diagnosis or it is possible that burnout might be overlooked on the account of these similarities, resulting in false treatments of the individuals who suffer from it.

Regarding the burnout-depression relationship, i.e., whether there is an overlap between burnout and depression, the results of our meta-analysis showed that there is an association between these two variables. Although burnout and depression are associated with each other, the effect size is not so strong that it would suggest they are the same construct. In other words, burnout and depression are more likely to be two different constructs rather than one. Additionally, as the MBI test was the one which was used in more of the half of the studies (55.1%), we examined the burnout–depression relationship in terms of context. According to our results, the studies that used the MBI test reported a lower association between burnout and depression compared to the studies that used other burnout measures, where the association between burnout and depression was higher.

Concerning the burnout—anxiety relationship, i.e., whether there is an overlap between burnout and anxiety, our results indicated a relationship between the two variables as well. In particular, although there appears to be an association between burnout and anxiety, this association is not so strong that it indicates an overlap between the two variables. This result indicates that although burnout is associated with anxiety, they are in fact different constructs. This finding can help us also answer the question of why some people develop burnout while others do not (Bühler and Land, 2003). According to our results, it is possible that individuals who are more prone to experiencing higher levels of anxiety (trait anxiety) are also more likely to develop burnout as well. As it regards the burnout-anxiety relationship in terms of context, likewise with the burnout-depression relationship, we found that in the studies that used the MBI test (63.9%) the effect between burnout and anxiety was lower compared to the ones that used a different burnout measure. However, it should be noted that there was variability in the inventories that were used in the research studies for assessing anxiety. It is possible that the effect sizes between burnout and anxiety would differ if there was a common widely used tool for assessing anxiety.

Overall, our results suggest that the studies that used measures other than the MBI burnout tool could potentially be artificially inflating the association between burnout and depression and burnout and anxiety as well. Burnout is an occupationally-specific dysphoria that is distinct from depression as a broadly based mental illness (Maslach et al., 2001). Maslach and Leiter (2016) have argued that while studies confirm that burnout and depression are not independent, claiming that they are simply the same mental illness is not supported by the accumulated evidence.

Another interesting finding of our meta-analysis was that the majority of the research studies that measured the relationship between burnout and depression, and burnout and anxiety, utilized cross- sectional designs (87% and 97% of the studies for depression and anxiety respectively).We noticed that there was a lack of longitudinal designs examining the burnout–depression and the burnout—anxiety relationship. Moreover, most of the longitudinal designs that were eligible for our meta-analysis did not examine directly the association between these two relationships, but they were focused mostly on whether burnout predicts depression or anxiety, or the opposite. As Bianchi et al. (2015b) aptly note, most longitudinal studies are not designed to examine casual relationships, but they mainly aim to investigate whether burnout can predict depression or vice versa. Therefore, although the burnout—depression and the burnout—anxiety relationships are found to be related, we are still not able to know whether these relationships are casual. Future studies need to focus more on utilizing longitudinal designs which will mostly aim at examining the causality of these relationships.

Overall, according to our results burnout and depression and burnout and anxiety appear to be different constructs that share some common characteristics and they probably develop in tandem, rather they fall into the same category with different names being used to describe them. However, further studies examining the psychosocial and neurobiological basis of these constructs are needed as well as their relationship with other illnesses (e.g., physical problems), as this field of research area is under investigated (Kaschka et al., 2011). It is worth noting that in their review Kaschka et al. (2011) mention that there appears to be a connection between burnout and cardiovascular musculoskeletal and cutaneous diseases and even with type II diabetes mellitus; and as burnout increases, the somatic co-morbidity appear to increase as well. Interestingly, a meta-analysis by Salvagioni et al. (2017) showed that burnout is a predictor of 12 somatic diseases, among which are; coronary heart disease, headaches, respiratory diseases and mortality under the age of 45 years old. Consequently, we can understand that burnout can have multifactorial psychological and somatic effects upon individuals.

Another field of research area, that would contribute further to the clarification of the association of these constructs, is the biological studies examining the neurobiological mechanisms behind burnout, depression, and anxiety. To this date such studies are scarce, however, researchers that have examined burnout, depression and anxiety at a biological level showed that these constructs appear to be similar. In particular, Korczak et al. (2010) found that neuroendocrine changes in individuals who suffer from burnout do not differ from the ones that suffer from depression or other stress related disorders; a finding that seems to be in accordance with Bakusic et al. (2017) review, as the authors suggest that burnout and depression share a common biological basis. Nevertheless, according to our meta-analysis results, burnout and depression and burnout and anxiety appear to be different rather the same constructs. These findings appear to be very crucial regarding medical diagnosis. Interestingly, only two European countries, i.e., Italy and Latvia, have recognized and classified burnout as an “occupational disease” (Eurofound, 2018), by distinguishing burnout from depression and anxiety, this will lead to more optimized practical implications to all experts who study burnout and work-stressors in general, and build more focused treatment plans.

All in all, we believe that our meta-analysis findings have helped toward the clarification of the relationship between burnout and depression, and burnout and anxiety. Additionally, another finding that emerged is that further studies need to be conducted which will be focusing on these two relationships and their behavioral, psychosomatic, and biological patterns as well. The findings of these studies will lead to the elucidation of the relationship of these constructs. Furthermore, it is worth mentioning that according to Eurofound (2018), during the past decade only a few countries across Europe have conducted surveys which focus exclusively on burnout and other European countries have mainly examined burnout related constructs, such as work stress and/or work-related exhaustion. Hence, we can realize that, besides the growing interest regarding the examination of both occupational work stressors and exhaustion and the clarification between burnout and depression and burnout and anxiety, more relevant studies are still lacking.

### Limitations

Our meta-analysis has some limitations. Firstly, we searched only for research studies that were conducted during the past decade. We cannot be certain if our results would be different if we were to include earlier studies as well. A second limitation is that the studies that did not provide appropriate statistical results were not included in the meta-analysis, therefore, again, we cannot be certain whether the inclusion of these studies would change our results. The problems of finding appropriate data to conduct analyses and the reluctance/inability of authors to provide such data when directly contacted has been identified within the literature as a significant barrier to conducting comprehensive meta-analysis (Hardwicke and Ioannidis, 2018). Lastly, a third limitation of our meta- analysis is that, although our database search was conducted through four well–known databases, we still cannot be certain whether all the studies that examined the burnout–depression and burnout–anxiety relationships are reported; a limitation which is also known as the “file–drawer problem” (Rosenthal, 1979). That is, we are not able to know if/and to what extent there was a selective publication bias and whether there were studies that remained unpublished due to non–statistically significant results. Hence, it is possible that studies in which these two relationships were examined but did not provide statistically significant results remained unpublished and, therefore, they were not included in our meta-analysis.

### Conclusions

In conclusion, our results showed that while there is statistical relationship between burnout and depression and burnout and anxiety, and while they are interconnected, they are not the same constructs. However, future studies examining these two relationships are still required in order to be able to draw safer conclusions. More longitudinal studies that focus on the causality of the burnout-depression and burnout—anxiety relationships are needed, as they will be able to clarify these two relationships. By conducting this meta-analysis, we aimed to examine the association between burnout and depression and burnout and anxiety. With our results we hope to inform potential effective interventions for treating burnout symptoms; by knowing the nature of a problem this can lead to more targeted solutions.

The modern workplace is characterized by significant proportions of people who feel exhausted, suffer from health problems, may be taking antidepressants or other medication, which can all contribute to feelings of diminished efficacy. The confluence of the aforementioned highlights the importance of clarifying the relationship between burnout and depression/anxiety, so as to avoid a one-dimensional approach to worker well-being.

## Author Contributions

PK developed and designed the methodology, conducted data collection, applied statistical techniques to analyze and synthesize the study data, prepared the published work, specifically writing the initial draft and acquired the financial support for the project leading to this publication. AM formulated the research goals and aims, developed and designed the methodology, applied statistical techniques to analyze and synthesize the study data, provided the analysis tool and prepared the published work, specifically with critical reviews, editing, and revisions. KG conducted data collection, applied statistical techniques to analyze and synthesize study data and prepared the published work, specifically with critical reviews, editing and revisions.

### Conflict of Interest Statement

The authors declare that the research was conducted in the absence of any commercial or financial relationships that could be construed as a potential conflict of interest.
